# Biochemical and Structural Insights into the Winged Helix Domain of P150, the Largest Subunit of the Chromatin Assembly Factor 1

**DOI:** 10.3390/ijms23042160

**Published:** 2022-02-15

**Authors:** Joëlle Ayoub, Martina Buonanno, Anna Di Fiore, Giuseppina De Simone, Simona Maria Monti

**Affiliations:** 1 Institute of Biostructures and Bioimaging, Consiglio Nazionale delle Ricerche (IBB-CNR), Via Mezzocannone, 16, 80134 Naples, Italy; ayoubjoelle@hotmail.com (J.A.); martina.buonanno@cnr.it (M.B.); anna.difiore@cnr.it (A.D.F.); 2 Department of Environmental, Biological and Pharmaceutical Sciences and Technologies, University of Campania Luigi Vanvitelli, 81100 Caserta, Italy; 3 Medical Laboratory-Molecular Biology, Saint George Hospital Medical Center, Rmeil-Youssef Sursok Street, Beirut 166378, Lebanon

**Keywords:** human P150, CAF-1, CHAF1A, DNA binding, EMSA, human diseases

## Abstract

The Chromatin Assembly Factor 1 is a heterotrimeric complex responsible for the nucleosome assembly during DNA replication and DNA repair. In humans, the largest subunit P150 is the major actor of this process. It has been recently considered as a tumor-associated protein due to its overexpression in many malignancies. Structural and functional studies targeting P150 are still limited and only scarce information about this subunit is currently available. Literature data and bioinformatics analysis assisted the identification of a stable DNA binding domain, encompassing residues from 721 to 860 of P150 within the full-length protein. This domain was recombinantly produced and in vitro investigated. An acidic region modulating its DNA binding ability was also identified and characterized. Results showed similarities and differences between the P150 and its yeast homologue, namely Cac-1, suggesting that, although sharing a common biological function, the two proteins may also possess different features.

## 1. Introduction

From yeast to human, genomic DNA is well packaged into a compact and ordered entity named nucleosome, the single unit of chromatin [1,2]. This repeating entity is formed by double-stranded DNA (145–147 bp) wrapped around an octamer of histones (a duplicate of H2A, H2B, H3, and H4) [3]. Nucleosomes undertake additional condensation stages to form the final level of compactness, leading to the chromatid of chromosome [4,5]. Despite the compactness, chromatin is known to be a dynamic structure able to unwrap during cellular processes (transcription, DNA replication, and repair) [6,7,8]. The transition between the wrapping and unwrapping state of the nucleosomes requires the involvement of different complexes known as histone chaperones [7]. Firstly described as preventers of histone–DNA aggregation, these proteins are able to bind histones, shield their positive charges, and avoid the unspecific DNA interactions [9,10,11]. More recently, histone chaperones were found to play an important role in guiding the specific nucleosome assembly path [12]. Most of them are conserved in eukaryotes and are classified based on their binding capacities (H3/H4 or H2A/H2B chaperones) [13,14]. Chromatin assembly reaction occurs in two steps: the addition of H3–H4 dimers onto the DNA occurs during the first step, while H2A–H2B dimers are added later on during the process [15,16]. One of the major actor orchestrating the addition of H3/H4 histones during DNA replication and repair is the Chromatin Assembly Factor 1 (CAF-1) [17,18,19,20], a three-subunit protein complex functionally conserved within eukaryotes [21,22,23]. The role of CAF-1 is not only restricted to nucleosome assembly, but also to chromatin silencing and heterochromatin integrity [16,24,25]. Due to its involvement into nuclear processes, CAF-1 affects cell fate decision and epigenetic control of gene expression [26]. In agreement with its role in DNA replication and repair, CAF-1 protein levels correlate with cell proliferation and cancer prognosis [27,28,29,30] and its dysregulation has been linked to human diseases [31,32]. 

CAF-1 has been found in *H. sapiens, D. melanogaster*, *M. musculus*, *C. elegans*, *S. cerevisiae*, and *A. Thaliana* and consists of three subunits in a 1:1:1 ratio in all these species [21,22,23,33,34]. In humans, the subunits forming CAF-1 were named according to the molecular weight based on their gel migration, i.e., P150, P60, and P48 [17], whereas the same counterparts in yeast were referred to as Cac-1, Cac-2, and Cac-3 (Cac, chromatin-assembly complex) [35]. 

**Figure 1:**
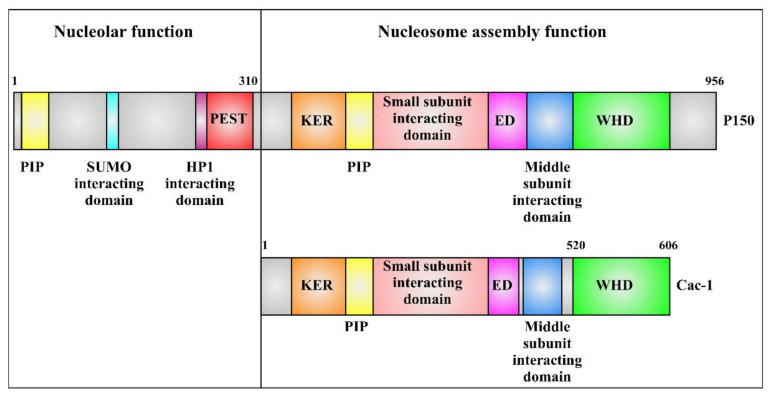
Schematic representation of P150 and Cac-1 components. The N-terminal region of P150 (residues 1–310) contains a PCNA interacting peptide (PIP) that has a strong activity in vitro, a SUMO protein interacting domain, an HP1 interacting region, and a PEST domain. The C-terminal region of P150 (residues 311–956) shows a domain organization similar to Cac-1. Both proteins contain a KER region, a PIP which is mainly responsible for maintaining the in vivo interaction with PCNA [40], the small subunit interacting domain, the ED sequence, the middle subunit interacting domain, and a WHD [43].

Among the three subunits, yeast Cac-1 has been the most investigated, and the reported results highlight its importance from a functional point of view. This protein, which consists of 606 residues, contains Cac-2 [36,37,38] and Cac-3 interacting domains [36,37,39], a proliferating cell nuclear antigen (PCNA) interacting region [40,41], in addition to DNA [36,42] and histone binding domains [36,38] (Figure 1). A first DNA binding domain, identified within a K/E/R rich domain (KER) [43], is located at the N-terminal part of the protein and predicted to adopt a coiled-coil structure. While no specific DNA sequence was identified for the binding, an optimal DNA length of at least 40 bp was suggested [43]. A second DNA binding domain, with a micromolar binding affinity, was identified in the C-terminal region of Cac-1, encompassing residues 520–606. This region was found to adopt a winged helix domain (WHD) structure characterized by four helices, two antiparallel strands, and a long wing loop. It has structural similarities with some WHD-containing proteins [44,45,46,47], but also some structural differences compared to canonical ones, such as the presence of a fourth helix [42]. Cac-1 WHD binds DNA in a sequence-independent manner consistently with its transitory functional role. Moreover, it binds different lengths of DNA through electrostatic interactions occurring between its positively charged residues and DNA phosphate groups [42]. Investigation on mutagenized WHD revealed its involvement, together with PCNA, in recruiting and stabilizing CAF-1 at the replication forks in yeast and mouse cells [42]. Finally, the histone binding domain, which contains part of an acidic stretch enriched in E/D (ED) amino acids [36,48], is involved in the interaction with histones [49,50]. Remarkably, this region has a regulatory mechanism on WHD, thus affecting the DNA binding ability of Cac-1 [38].

Here, we focused our attention on P150 (also referred as CHAF1A), the largest subunit of the human CAF-1 complex. This protein was first cloned by Kaufman and coworkers in 1995 [51] and later sequenced by Dong and coworkers [52]. It consists of 956 amino acids and is subject to phosphorylation [53]. Similarly to the yeast homologue, P150 has a leading role in nucleosome assembly as highlighted upon depletion and mutation experiments [26]. From a functional point of view, the P150 sequence can be divided into two main regions: the N-terminal one, encompassing residues 1 to 310 which is involved in nucleolar functions [27], and the region encompassing residues 311 to 956, which is necessary and sufficient for nucleosome assembly [51] (Figure 1). P150 N-terminal region is necessary for the localization of different proteins such as nucleophosmin and nucleolin, and its loss is lethal in vivo [27]. It contains four major identified regions: a PEST domain [51,54], three domains interacting respectively with HP1 (Heterochromatin Protein 1) [55,56,57], SUMO proteins (Small Ubiquitin-like MOdifier) [26,58], and PCNA (proliferating cell nuclear antigen) [59] (Figure 1). Notably, residues 1–310 and 855–956 corresponding to the N- and C- terminal parts of P150 are absent in Cac-1. On the contrary, the region 311–854 of P150 is conserved in the yeast homologue, where it maintains the same function [51]. Differently from Cac-1, for which many studies have been so far carried out [9,60,61,62], structural and functional studies targeting P150 are still very limited. However, most of the described Cac-1 regions were conserved in P150, allowing the identification of the homologous domains in the human protein (Figure 1) [43]. 

Recently, Zhang and coworkers identified a DNA-binding domain in human and mouse P150, corresponding to the Cac-1 WHD, and proved its ability to bind 58 dsDNA [42]. Here, following a multidisciplinary approach, we further investigated this domain and identified a regulatory region of its DNA binding activity. Results highlighted both similarities and differences between the P150 and Cac-1, suggesting that, although sharing a common biological function, the two proteins could adopt a different mechanism of action. 

## 2. Results

### 2.1. Design and Chemico-Physical Characterization of P150 WHD Domain

**Figure 2:**
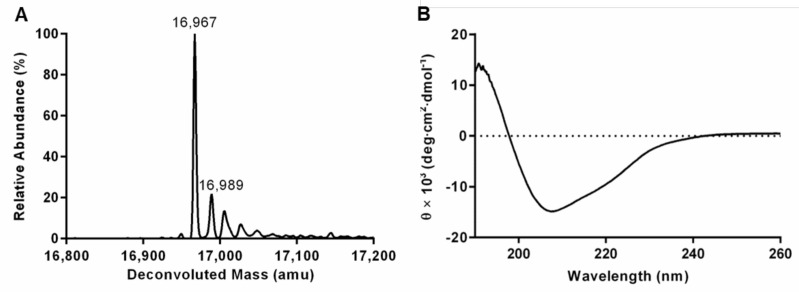
Biochemical features of P150_721–860._ (**A**) Deconvoluted mass spectrum of P150_721–860_. The experimental molecular weight corresponds to the polypeptide lacking the initial methionine and comprehensive of the (His)_6_-tag. (**B**) Far-UV CD spectrum of P150_721–860_ recorded in 1 mM Tris-HCl, pH 7.5, at a protein concentration of 7 µM at 20 °C.

Zhang and coworkers identified the region 727–854 of human P150 as the one corresponding to Cac-1 WHD [42]. Interestingly, Phyre 2 [63], an online software able to predict and analyze protein 3D structures, identified residues 723–824 of P150 as belonging to a stable WHD similar to the one present in the Cockayne syndrome group B protein 1 [64]. Taking together these findings, in order to cover the whole P150 WHD, the protein region encompassing residues 721–860 (hereafter referred as P150_721–860_) was designed, cloned, expressed, and subsequently purified (Figure S1A). Briefly, P150_721–860_ gene was cloned into pETM13 vector, allowing the expression of the protein with a His tag at its C-terminal part. Optimized expression in LB was achieved in *E. coli* BL21(DE3)pLysS strain at 16 °C with 1 mM isopropyl-thio-D-glactosidase (IPTG). Upon three steps of purification, a purity level greater than 95% with a final yield of 2.5 mg/L of growth medium was achieved. Mass spectrometry analyses were carried out to confirm protein identity and purity. Results (Figure 2A) showed one main peak at 16,967 Da corresponding to P150_721–860_ lacking the starting N-terminal methionine (MW_theoretical_ = 16,967 Da). This excision, due to the methionylamino peptidase [65], is a widely described phenomenon for recombinant proteins expressed in *E. coli* [66,67].

The secondary structure content of P150_721–860_ was assessed by circular dichroism (CD). Results showed negative molar ellipticity values at 208 nm and 222 nm in addition to a positive ellipticity below 198 nm (Figure 2B) indicative of the presence of 𝛼-helices. The reduced magnitude of the negative ellipticity at 222 nm highlighted the contribution of β-sheets [68], thus suggesting the occurrence of both 𝛼-helical and β-sheet secondary structures, as expected for WHD proteins [42,69]. Thermal stability was assessed by CD following changes in ellipticity at 222 nm when heating up the protein from 20 °C to 90 °C. Experiments revealed a melting point at 40 °C (Figure S2A). Notably, the secondary structure of P150_721–860_ was almost fully recovered once the sample returned to its initial temperature (Figure S2B). 

**Figure 3:**
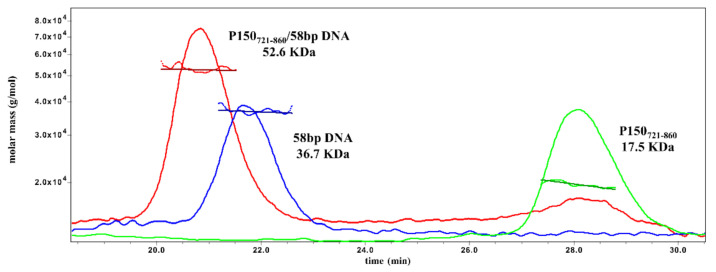
Molar mass of P150_721–860_, ds58 DNA, and P150_721–860_ /ds58 DNA complex by SEC-MALS-QELS analysis. Overlay of light scattering chromatograms of recombinant P150_721–860_ (green line), ds58 DNA (blue line), and P150_721–860_/ds58 DNA complex (red line) samples. The molar mass of each sample is reported. Runs were performed in 20 mM Tris-HCl, 150 mM NaCl, pH 7.5.

The quaternary structure of P150_721–860_ was investigated by SEC-MALS-QELS [70], revealing that in solution, the protein is a monomer with a molecular weight of 17.5 KDa (±0.1%) (Figure 3) in agreement with previously reported results by Mattiroli and coworkers for the isolated Cac-1 WHD [38]. On the contrary, Liu and coworkers showed, by fluorescence anisotropy, that Cac-1 forms a dimer in solution through the C-terminus experiments, and highlighted the influence of buffer composition on the oligomerization state of the protein [48]. Thus, to explore putative effects of ionic strengths on the quaternary structure of P150_721–860_, LS analyses were carried out at different concentration of NaCl [71,72]. Our results showed that either at 150 mM or 500 mM NaCl, P150_721–860_ is monomeric (Figure S3). 

### 2.2. P150_721–860_ Binds dsDNA

Electrophoretic mobility shift assay (EMSA) was carried out to explore the propensity of P150_721–860_ to bind DNA. Firstly, we investigated whether P150_721–860_ showed preferences in binding DNA with different base composition (AT- or GC-rich) or length (16 and 58 dsDNA). Obtained results highlighted that P150_721–860_ is able to bind dsDNA no matter the composition (Figure S4) and length (Figure S5), consistently with previously reported results performed on Cac-1 [42]. 

**Figure 4:**
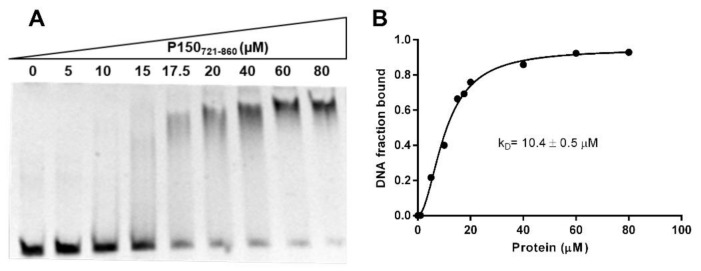
DNA binding ability of P150_721–860_ to 58 bp dsDNA assessed by EMSA. (**A**) 1 µM 58 bp dsDNA was incubated with increasing concentration of P150_721–860_. Gel was stained with Sybr Gold prior to visualization with UV light. (**B**) The density value of each band quantified by ImageJ was analyzed by Graph Pad-Prism, Version 7.

Subsequently, by fitting the data using a nonlinear regression, a K_D_ value of 10.4 ± 0.5 µM was estimated (Figure 4), in agreement with the DNA dissociation constant determined for Cac-1 WHD [42]. The stoichiometry of binding was evaluated by light scattering experiments. Upon incubation of an excess of P150_721–860_ over 58 bp dsDNA (molar ratio of 3.2:1, P150_721–860_: dsDNA 58 bp), a complex in a 1:1 molar ratio was clearly identified (Figure 3). 

### 2.3. ED Domain Modulates DNA Binding to P150_721–860_

In yeast, the ED region enriched in glutamic and aspartic acids constitutes part of the histone binding interface of Cac-1 [38,43,48]. In absence of histones, the acidic residues of this region make intramolecular interactions with the basic residues of WHD, masking the DNA binding activity of CAF-1 [36,38]. As revealed by inspecting the sequence, this acidic region is also present in P150 located at the N-terminal part of the WHD here investigated (Figure 1). With the aim to explore whether the described inhibitory mechanism is preserved in P150, a new construct was designed extending the length of P150_721–860_ at the N-terminal region to incorporate the acidic domain. Accordingly, a new construct, hereby named as P150_575–860_, was obtained cloning residues from 575 to 860 of P150 in pETM13 (Figure S1B). The recombinant protein was successfully produced in *E. coli*, purified, and investigated for its ability to bind DNA. EMSA was carried out incubating the same quantities of 58 bp dsDNA with increased quantities of P150_575–860_. Results showed that no complex formation was detected, highlighting that, despite the presence of the WHD, the DNA binding activity of P150 is hindered by the presence of the ED stretch. These data confirm that, similarly to what was observed for Cac-1, in the human subunit, the ED domain modulates the DNA binding activity of WHD (Figure S6). 

## 3. Discussion

During DNA replication and DNA repair, the histone chaperone CAF-1 actively orchestrates nucleosome assembly, interacting with replisome and depositing H3–H4 directly onto newly synthetized DNA [73,74]. CAF-1 plays pivotal roles in maintaining genome stability; thus, any dysregulation of the complex and its subunits can likely cause alteration in the genome. Nevertheless, biochemical and structural studies on the proteins of the complex are rather limited, with few functional studies carried out only recently [36,38,42]. These investigations led to the development of models suggesting how the three different subunits work in the assembly. Notably, most of these studies have been carried out on *S. cerevisiae* proteins and results were then translated to the human system [36,38,42,43].

Here, we report the first detailed biochemical and structural characterization of a P150 DNA binding region and its regulatory mechanism. Starting from preliminary data previously obtained by Liu’s group [42], we expressed and purified the putative WHD of P150, namely P150_721–860_. We demonstrated, by light scattering experiments, that in solution, P150_721–860_ keeps a monomeric structure independently of ion strength. Literature data on this point are rather controversial; indeed, Mattiroli and coworkers, in agreement with our results, reported a monomeric structure for the isolated Cac-1 WHD [38], whereas the crystallographic structure of the same protein determined by Liu and coworkers highlighted a dimeric arrangement [48]. Interestingly, the same authors reported that homodimerization does not occur when changing buffer composition [48].

**Figure 5:**
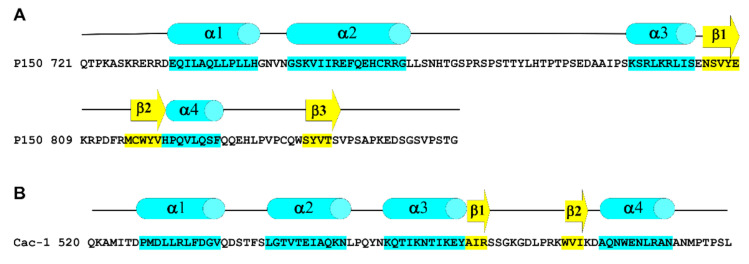
Schematic representation of P150 and Cac-1 WHD secondary structure. Helices and strands of P150 WHD (**A**) have been predicted by the PHD program, whereas those of Cac-1 WHD (**B**) have been calculated with Whatcheck [78] using the crystallographic coordinates (PDB accession code 5JBM) [48]. Helices and strands are represented as cyan cylinders and yellow arrows, respectively. Primary sequences are also shown.

Consistently with its biological role, P150_721–860_ binds DNA with different base composition (AT- and GC-rich) and lengths (dsDNA 16 and 58 bp) with a binding affinity in the micromolar range, in accordance with values already reported for WH proteins such as isolated Cac-1 (K_D_ = 2 µM) [42], Ash2L (K_D_ = 12 µM) [75], FoxM1 (K_D_ = 7 µM) [76], and Rtt106 (K_D_ > 20 µM) [77]. The absence of a sequence specificity and the low DNA binding affinity are in line with the necessity of a conserved mechanism of CAF-1 to bind replicated or repaired DNA rather than specific DNA sequences [42]. Binding stoichiometry between P150_721–860_ and 58 bp dsDNA was evaluated in vitro giving a 1:1 molar ratio, in contrast to that observed for Cac-1, which binds an 18 bp dsDNA in a molar ratio of 2:1 (Cac-1:DNA) [38]. Taken together, these findings suggest that WHD from human and yeast may share similarities and differences, as revealed also by the structural comparison of the two proteins. Indeed, a secondary structure prediction of P150_721–860_, carried out with the online program PHD (accessed date 10 December 2021, https://npsa-prabi.ibcp.fr/cgi-bin/npsa_automat.pl?page = /NPSA/npsa_phd.html), reveals that, although this protein retains the typical elements of the WHD present in Cac-1, significant differences are also present. In particular, P150_721–860_ contains a big insertion between predicted helices α2 and α3 with respect to Cac-1 WHD; moreover, it is longer and contains, at the C-terminus, an additional coil region followed by a β-strand (Figure 5). The observed biochemical and structural differences between the human and the yeast protein could also reflect variations in the mechanism of action, but this topic needs to be further investigated. In this context, it is also worth noting that the full-length P150 contains an additional sequence (residues 861–956) whose function has to be completely unveiled (Figure 1). 

The binding ability of P150_721–860_ to DNA is modulated by an acidic region which precedes the WHD. Accordingly, an enlarged construct comprehensive of the ED-rich region, namely P150_575–860_, is not able to bind DNA. It is assumed that the negatively charged residues of the ED domain sequestrate the WHD, making it inaccessible to DNA binding [43]. Similarly to what happens in yeast, it can be hypothesized that in the whole human CAF-1 complex, the presence of H3–H4 histones, which engages the acidic ED domain, makes the WHD free to interact with DNA [43]. 

In conclusion, we investigated, for the first time, the biochemical and structural features of the P150_721–860_ DNA binding region. Results indicated the typical features of a WHD containing both alpha and beta secondary structure elements. P150_721–860_ directly interacts with dsDNA [43] in vitro and its binding is modulated by the presence of an acidic stretch encompassing region 575–720. The detected protein/DNA binding ratio is 1:1, different from what was observed in yeast, and this could be indicative of a different mechanism of action between the human and the yeast protein in vivo. This hypothesis is supported by the presence in P150 of a C-terminal region which is completely absent in the yeast homologue and whose role still needs to be defined. Further experiments are currently underway in our lab to provide insights into this C-terminal region 

## 4. Materials and Methods

### 4.1. P150_721–860_ and P150_575–860_ Design, Cloning, Expression, and Purification

P150_721–860_ and P150_575–860_ cDNAs (GeneArt, ThermoFisher Scientific), optimized for expression in *E. coli,* were amplified by PCR using forward and reverse primers as listed in Table 1. Amplified cDNAs were cloned into pETM13 vector (kind gift from EMBL Heidelberg) using *NcoI* and *XhoI* enzymes (New England Biolabs, NEB). This vector was chosen for its feature to encode a six-histidine tag (His-tag) at the C-terminus. The integrity of the sequence was assessed by DNA sequencing upon appropriate digestion with restriction enzymes.

Each recombinant protein (P150_721–860_ or P150_575–860_) was expressed by transforming *E. coli* BL21(DE3)pLysS strain (kind gift from EMBL Heidelberg) with the corresponding plasmid. Cells were grown in LB and induced with 1 mM IPTG at 16 °C or 22 °C for the expression of P150_721–860_ and P150_575–860_, respectively. After an overnight culture, cells were pelleted by centrifugation (7500 rpm for 20 min at 4 °C). Identically for both proteins, pellets were resuspended in 20 mM Tris-HCl, 500 mM NaCl, pH 7.5 supplemented with PMSF (1 mM), DNaseI (5 µg/mL), lysozyme (0.1 mg/mL), and 1 μg/mL of the following protease inhibitors (aprotinin, leupeptin, and pepstatin). Cells were then sonicated on ice and centrifuged (13,500 rpm for 20 min at 4 °C). Supernatant was purified at room temperature on ÄKTA FPLC (GE Healthcare). P150_721–860_ was purified by two affinity chromatography steps (TALON and heparin) and a Superdex 75 size exclusion chromatography (SEC), while P150_575–860_ purification consisted of a TALON affinity chromatography and SEC. Protein quality was assessed by 20% SDS-PAGE and ESI-TOF MS analysis (Agilent Technologies, Cernusco Sul Naviglio, Italy). Deconvolution was carried out by means of the Agilent MassHunter Qualitative software.

### 4.2. ESI–TOF-MS Analyses

A C4 Biobasic 50 × 2.1 mm ID column (ThermoFisher Monza, Italy) was used to load P150_721–860_ and P150_575–860_ proteins with a flowrate of 0.2 mL/min. ESI–TOF-MS analyses were carried out on an Agilent 1290 Infinity LC System coupled to an Agilent 6230 time-of-flight (TOF) LC/MS system (Agilent Technologies, Cernusco Sul Naviglio, Italy). The LC module Agilent 1290 was coupled to a photodiode array (PDA) detector and a 6230 time-of-flight MS detector, along with a binary solvent pump degasser, column heater, and autosampler. Chromatographic separation was performed using, as solvent A, 0.01% TFA in H_2_O (*v*/*v*), and as solvent B, 0.01% TFA in CH_3_CN (*v*/*v*). A fully reduced sample with 10 mM DTT was also analyzed. Deconvolution was carried out by means of the Agilent MassHunter Qualitative software.

### 4.3. Circular Dichroism 

Circular dichroism (CD) experiments were carried out as previously described [79,80]. Spectra were recorded using a Jasco J-815 spectropolarimeter (Jasco, Essex, UK), equipped with a Peltier temperature control system in the far-UV range 190–260 nm. Measurements were run at 20 °C with three accumulations, using a 1 mm quartz cell. Experiments were performed using 7 µM of P150_721–860_ in 1 mM Tris-HCl, pH 7.5. Raw spectra were corrected for buffer contribution, converted to mean molar ellipticity per residue (θ) (deg cm^2^ dmol^−1^), and visualized using GraphPad software. For thermal stability experiments, P150_721–860_ was heated from 20 to 100 °C with a temperature increase of 1 °C/min and spectrum recorded at 222 nm, as above described. The molar ellipticity values at 222 nm were plotted as function of the temperature using GraphPad software. Additionally, the three spectra (5 °C, 90 °C, and 5 °C after heating) were recorded as previously described once the fixed temperature was reached within ± 0.1 °C set by a Peltier device. 

### 4.4. Light Scattering

SEC–MALS–QELS (size exclusion chromatography–multi-angle light-scattering–quasi-elastic light scattering) analyses were carried out as previously described [81]. Briefly, the sample was loaded on a Superdex 75 10/300 GL (GE Healthcare) mounted in line with a multi-angle detector (mini-DAWN TREOS, Wyatt Technology) and a refraction index detector (Shodex RI 101). The first two runs were performed by injecting either P150_721–860_ or 58 dsDNA, 475 µg and 330 µg, respectively. The third run consisted of mixing together the previously mentioned quantities achieving a 3.2:1 molar ratio (protein: DNA) incubating the mixture on ice for 45 min before loading the column. Runs were performed in 20 mM Tris-HCl, 150 mM NaCl, and pH 7.5 at room temperature. When specified, LS was carried out in 20 mM Tris-HCl, 500 mM NaCl, pH 7.5. Data analysis was carried out using ASTRA 5.3.4.14 software (Wyatt Technology Corporation). 

### 4.5. Electrophoretic Mobility Shift Assay 

The 16 and 58 bp oligonucleotides (Table 2) used for binding assays were from Stazione Zoologica Anton Dohrn (Naples, Italy). Two strands of complementary ssDNA were annealed in 10 mM Tris-HCl, 50 mM NaCl, 1 mM EDTA, and Ph 7.5 buffer to form dsDNA. Protein dilutions were mixed with dsDNA in 20 Mm Tris-HCl, 100 Mm NaCl, 1 Mm DTT, and pH 7.5. In detail, when staining with ethidium bromide (EtBr) (Sigma), 21 µM of 16 pb dsDNA were mixed with P150_721–860_ in 0–15 molar ratio. Similar quantities were used in the AT- and GC-rich binding experiments. In the case of 58 dsDNA, 2 µM of DNA were mixed with P150_721–860_ or P150_575–860_ in 0–25 molar ratio. When staining with Sybr Gold, 1 µM of dsDNA was mixed with P150_721–860_ in 0–80 molar ratio. Binding reaction was incubated 45 min on ice. Gel loading dye, purple (6X, no SDS, New England Biolabs) and glycerol (final concentration of 5%), were added to the mixture, prior to the loading into a 6% PAGE pre-run in 0.5X TBE buffer. The pre-run and the run were performed at 75 V on ice, for 30 and 45 min, respectively. Gels were stained either with ethidium bromide (EtBr) or with Sybr Gold (Thermofisher) prior to visualization with UV light. Each fraction bound was calculated by quantifying the density of each lane by ImageJ [82]. The obtained data were analyzed by nonlinear regression equation with hill slope using the GraphPad Software. Results come from at least three experiments.

## Figures and Tables

**Table 1:** List of the primers with the restriction site underlined.

Name of the Construct	Primers (F: Forward; R: Reverse)	
P150_721–860_	F	CGCGCGCCATGGGCCAGACCCCGAAAGCAAGC
	R	CGCGCGCTCGAGACCGGTGCTCGGAACG
P150_575–860_	F	CGCGCGCCATGGGCAACAAAAAAACCGCACTGATTCGTG
	R	CGCGCGCTCGAGACCGGTGCTCGGAACG

**Table 2:** List of single-strand DNA sequences.

DNA Length	Sequence (5′-3′)
16 bp	TGCTACCGATCGATAC
16 bp GC rich	ATCGCCCGCGCACGCA
16 bp AT rich	ATAATCGATATTCGTT
58 bp	ATACATTTTATGACTGGAAACTTTTTTGTACAA CACTCCAATAAACATTTTGATTTTA

## Supplementary Materials

The following supporting information can be downloaded at: https://www.mdpi.com/article/10.3390/ijms23042160/s1.
