# Supplementary figures and images for: Biochemical and Structural Insights into the Winged Helix Domain of P150, the Largest Subunit of the Chromatin Assembly Factor 1
Source: Int J Mol Sci. 2022 Feb 15;23(4):2160. doi: 10.3390/ijms23042160 (PMC8874411; doi:10.3390/ijms23042160)

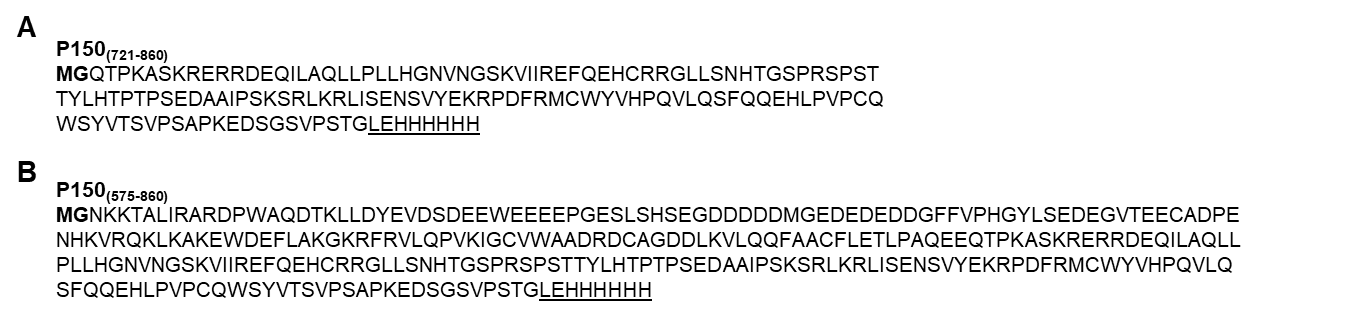

Supplement: Supplementary file 1 [file ijms-23-02160-s001.zip › ijms-1572763-supplementary/Additional file 1/Figure_S1A_1B.tif]

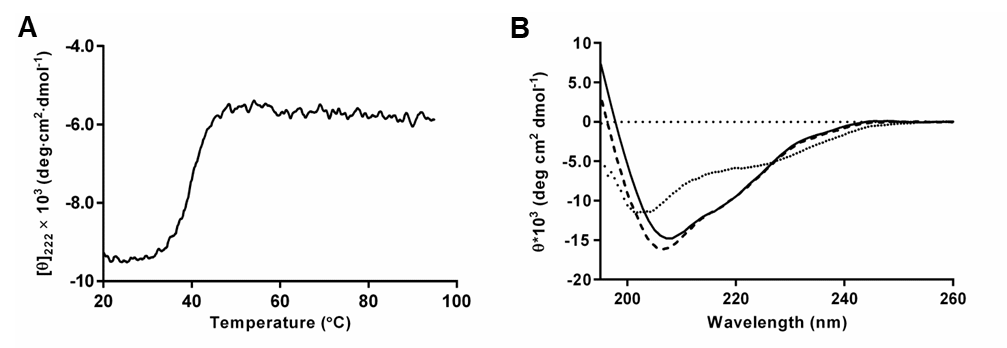

Supplement: Supplementary file 1 [file ijms-23-02160-s001.zip › ijms-1572763-supplementary/Additional file 1/Figure_S2A_S2B.tif]

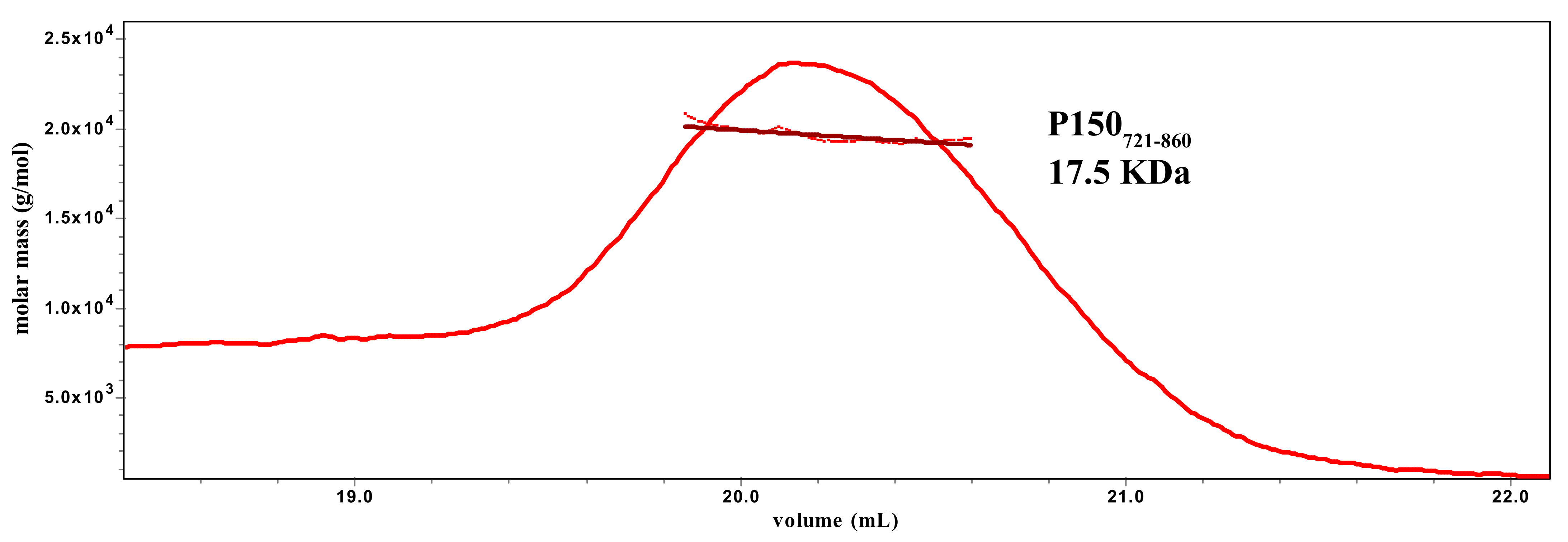

Supplement: Supplementary file 1 [file ijms-23-02160-s001.zip › ijms-1572763-supplementary/Additional file 1/Figure_S3.tif]

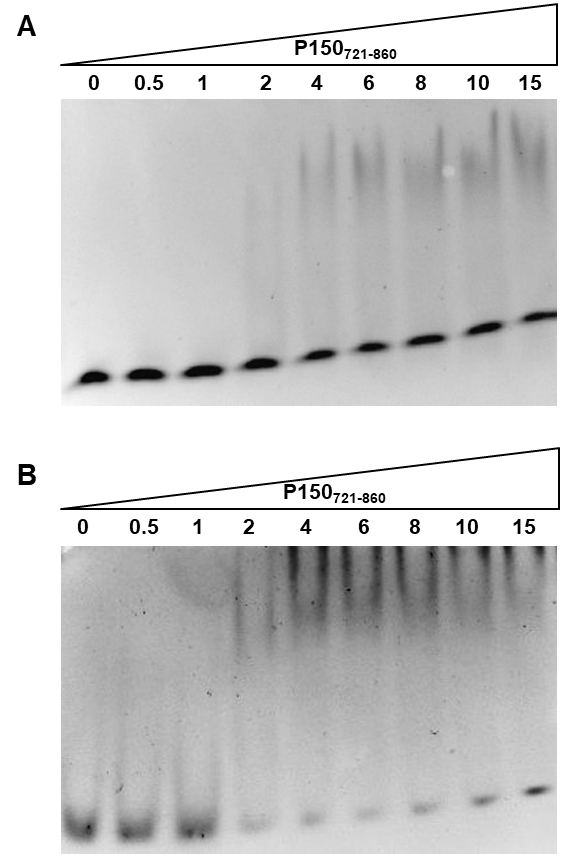

Supplement: Supplementary file 1 [file ijms-23-02160-s001.zip › ijms-1572763-supplementary/Additional file 1/Figure_S4A_S4B.tif]

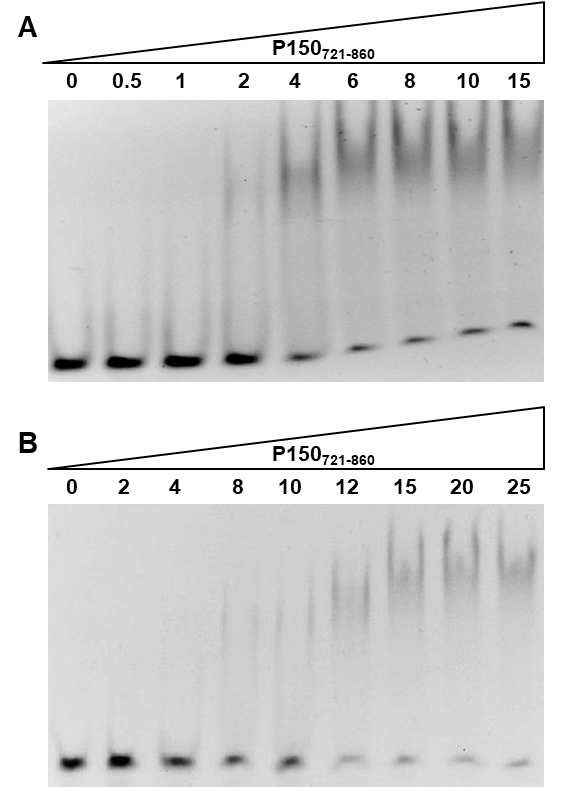

Supplement: Supplementary file 1 [file ijms-23-02160-s001.zip › ijms-1572763-supplementary/Additional file 1/Figure_S5A_S5B.tif]

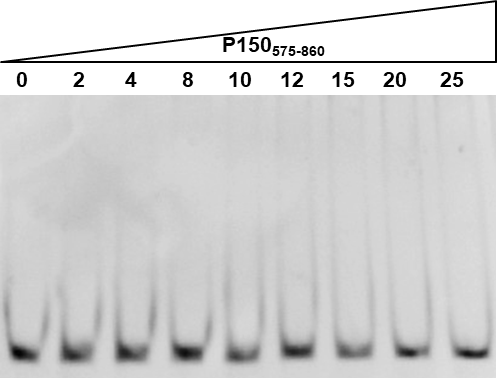

Supplement: Supplementary file 1 [file ijms-23-02160-s001.zip › ijms-1572763-supplementary/Additional file 1/Figure_S6.tif]
